# Center Deviation Measurement of Color Contact Lenses Based on a Deep Learning Model and Hough Circle Transform

**DOI:** 10.3390/s23146533

**Published:** 2023-07-19

**Authors:** Gi-nam Kim, Sung-hoon Kim, In Joo, Gui-bae Kim, Kwan-hee Yoo

**Affiliations:** 1 Department of Computer Science, Chungbuk National University, Cheongju 28644, Republic of Korea; kgn4192@chungbuk.ac.kr (G.-n.K.); sidsid84@chungbuk.ac.kr (S.-h.K.); jooin95@chungbuk.ac.kr (I.J.); 2 NeoVision Co., Ltd., Cheongju 28644, Republic of Korea; kimggbb@gmail.com

**Keywords:** computer vision, image segmentation, DeepLabV3+, Hough circle transform, deep learning, data augmentation, color contact lens, center deviation

## Abstract

Ensuring the quality of color contact lenses is vital, particularly in detecting defects during their production since they are directly worn on the eyes. One significant defect is the “center deviation (CD) defect”, where the colored area (CA) deviates from the center point. Measuring the extent of deviation of the CA from the center point is necessary to detect these CD defects. In this study, we propose a method that utilizes image processing and analysis techniques for detecting such defects. Our approach involves employing semantic segmentation to simplify the image and reduce noise interference and utilizing the Hough circle transform algorithm to measure the deviation of the center point of the CA in color contact lenses. Experimental results demonstrated that our proposed method achieved a 71.2% reduction in error compared with existing research methods.

## 1. Introduction

With the recent emphasis on digital transformation (DX) following the fourth industrial revolution [1], new approaches and changes are taking place via the construction of smart factories. Particularly, various defects arising from image-based products are gaining attention as representative technologies [2]. Notable related studies include a deep learning-based auto-sorting system by Wang et al. [3], Convolutional Neural Network (CNN)-based [4] defect inspection by Ha et al. [5], shipyard painting defect detection by Ma et al. [6], and steel-plate surface defect detection by Sharma et al. [7]. Similar defects can also occur in the production process of color contact lenses. Given that contact lenses are directly attached to the eyes, ensuring their quality is highly important [8]. However, research on defect detection during the production process remains insufficient. Therefore, this study aims to propose a method for detecting defects in contact lenses.

**Figure 1:**
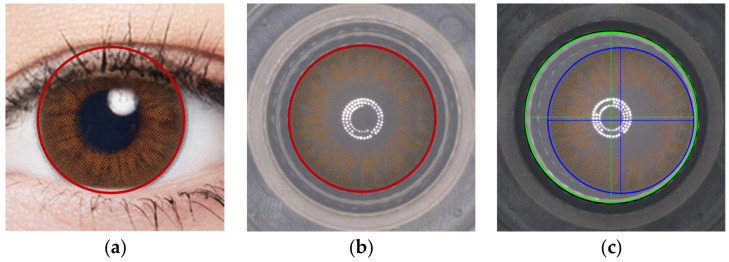
(**a**) An eye with a color contact lens, in (**b**), the area inside the red circle is the colored area (CA) of a color contact lens, and (**c**) possible center deviation (CD) defects that can occur during the manufacturing process. The figure demonstrates a CD defect that is skewed to the bottom right corner. In (**c**), the blue circle represents the CA, and the green circle represents the frame area (FA), which serves as a reference for measuring the displacement of the center point of the CA.

In this study, we discuss the injection molding [9] process of color contact lenses produced using the sandwich method [10,11]. During this process, the colored area (CA), which provides a cosmetic effect to the eye, is printed on the contact lens (as shown in Figure 1a,b). However, various defects can occur simultaneously in the printing process [12]. One such phenomenon is “center deviation (CD) defects”, as shown in Figure 1c, where the CA deviates from the center point of the frame. Other defects include “colored defects” (incomplete coloration), “line defects” (scratches), “partial design defects” (misprinted pattern parts), and “dot defects” (missing dots within the CA). We aimed to propose an accurate and efficient method for measuring the degree of CD of the CA in color contact lenses, as shown in Figure 1c. To achieve this, we utilized the semantic segmentation model [13] and the Hough circle transform (HCT) [14], which are tailored to the unique characteristics of color contact lenses, and detected defects based on this deviation.

Our proposed method aims to enhance production efficiency and product quality by detecting defects in advance and enabling precise adjustments in the production machinery based on the measured CD. This solves the CD defect problem in the manufacturing process of color contact lenses, thus ensuring the production of safer and higher-quality products. The results of this study can be utilized not only in the color contact lens industry but also in defect detection and quality control in various manufacturing industries.

In real-world applications, on-site workers need to assess the degree of deviation in the printed CA and adjust the machine printing of the CA accordingly. They also need to understand the causes of success or failure in detecting the CD defects for rapid problem identification. Therefore, this study employed a semantic segmentation model as an intermediate step in image analysis. Through the model’s predicted results, we assessed the degree of CD defects using the HCT. Although this method hinders a direct understanding of how deep learning predicts, it enables an indirect comprehension of the CD defects through the visualized outputs of DeepLabV3+. Subsequently, the HCT is employed to provide a quantitative assessment of the degree of CD.

**Figure 2:**
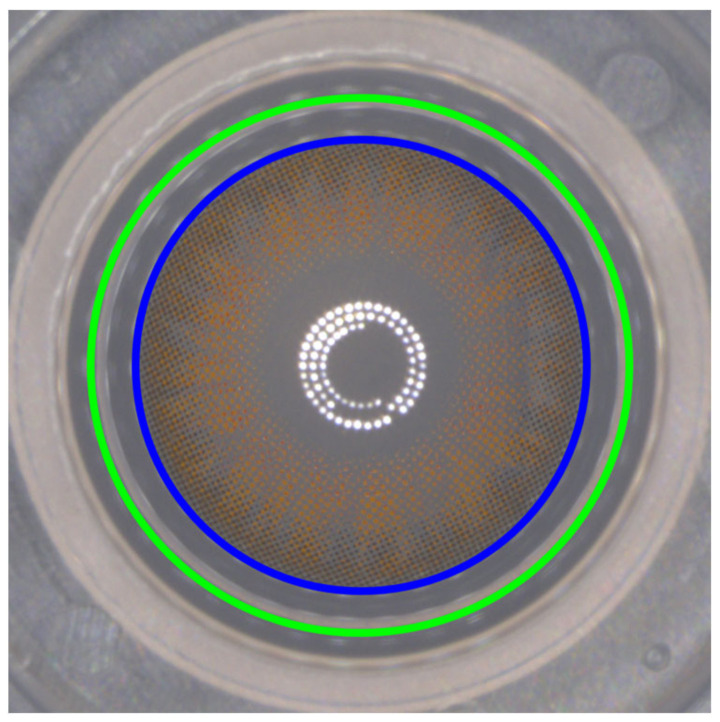
The blue and green circles indicate the boundaries of CA and FA, respectively. In this figure, the blue circle represents the CA, and the green circle represents the frame area (FA), which serves as a reference for measuring the displacement of the center point of the CA.

To accurately measure the center point deviation of the color contact lens, a specific area within the image is initially defined. The evaluation of the color contact lens manufacturing process relies on the distance between the centers of two crucial circles: the CA and frame area (FA), as depicted in Figure 2. In this method, rather than relying on the center of the acquired image, the center coordinates of FA, represented by the green circle, are preferred as the reference point for measuring the deviation distance of the CA as the image capture may not always accurately capture the center of the lens. This approach ensures precise measurements and evaluations, facilitating a more reliable assessment of lens quality.

## 2. Related Work

Previous studies, such as those conducted by Raghavendra et al. [15] and Choudhary et al. [16], have primarily focused on detecting and classifying contact lenses, determining whether they are present, and categorizing them accordingly. Other studies, such as Kimura et al. [17] and Parzianello et al. [18], researched iris recognition while contact lenses were worn.

Kim et al. [12] and Kim et al. [19] discussed the defect detection in color contact lenses from the injection molding process. However, these studies focused only on classifying the presence of defects, not on measuring the deviation distance of the center point. Another study by Kim et al. [20] presented a method for measuring the deviation distance of the center point using HCT.

**Figure 3:**
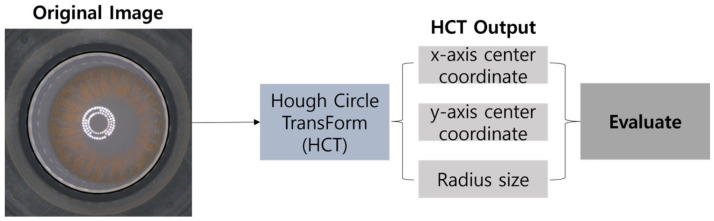
Overall flow diagram of the research method by Kim et al. [20].

As shown in Figure 3, Kim et al. [20] proposed a method for center deviation measurement using HCT, which is one of the famous traditional techniques in image processing. However, the method has certain limitations that can be susceptible to noise caused by variations in brightness from surrounding environments, changes in lens types, and incomplete image data or edge detectors [21]. And also, it can lead to the omission of curves or the occurrence of false data pixels, requiring HCT parameter adjustments to address these issues.

Therefore, this paper proposes a method that can handle variations in shooting environments and lens types when measuring the deviation distance of the center point in color contact lenses. Additionally, we propose a method to classify defects based on the measured distance. The proposed method employs a semantic segmentation model to simplify images and resolve errors caused by noise during circle detection using HCT.

## 3. Proposed Method for Computing Center Deviation (CD) of Color Contact Lenses

**Figure 4:**
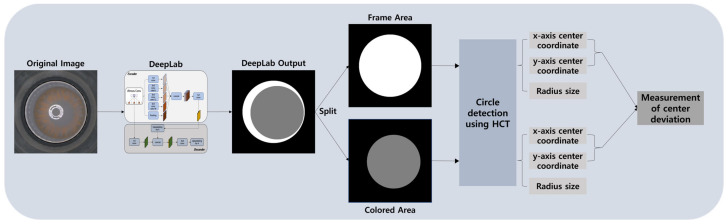
Overall flow diagram of the proposed method.

In this section, we present our proposed method for computing the two circles, CA and FA, as shown in Figure 4. First, we utilize DeepLabV3+ [22], a semantic segmentation model, to segment the predicted results into the FA and CA. Next, the segmented image is split into separate images for CA and FA. The HCT algorithm is then applied to each of these split areas to calculate the center coordinates and the radii of the circles for FA and CA. Finally, using the calculated center coordinates, we measure the deviation distance between the centers. The following subsections provide further details of this process.

### 3.1. Semantic Segmentation of Color Contact Lenses Using DeepLabV3+

**Figure 5:**
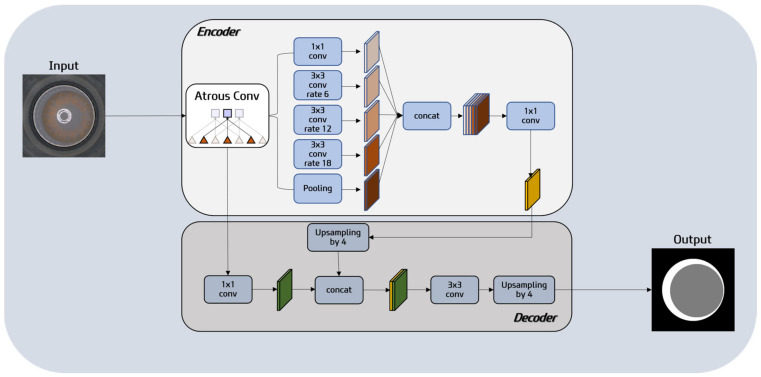
DeepLabV3+ model structure for segmenting FA and CA from an original color contact lens image.

**Figure 6:**
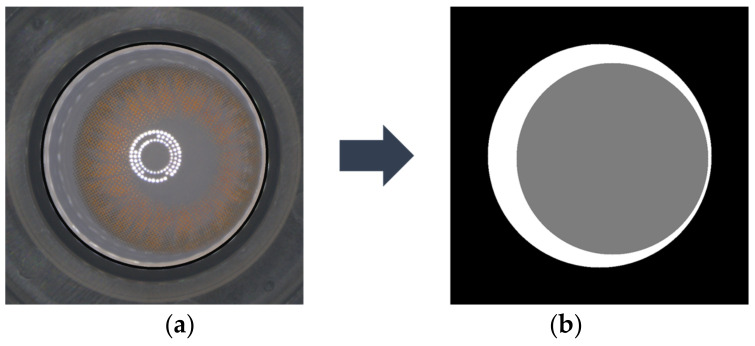
Original color contact lens image (**a**); ground truth image for segmentation of FA and CA (**b**).

Calculating CA and FA in color contact lens images was performed by applying DeepLabV3+ as shown in Figure 5. DeepLabV3+ is a semantic segmentation model that combines fully convolutional networks (FCNs) [23] and residual networks [24], enabling pixel-level classification of input images. In this study, DeepLabV3+ is utilized to perform pixel-wise segmentation of the lens image into the background, CA, and FA. As shown in Figure 6, the pre-trained DeepLabV3+ model receives an image like that shown in Figure 6a and predicts the image as shown in Figure 6b. The semantic segmentation result shows black, white, and gray parts, where gray (white) represents CA (resp. FA). This segmentation process ensures a more accurate localization of the circles by reducing interference due to noise and minimizing computational complexity in the HCT process. Such segmentation enhances the overall precision in determining the positions of the circles compared with direct processing of the original image. The basic architecture of DeepLabV3+ is as follows:

Encoder: The encoder in DeepLabV3+ is a pivotal component responsible for generating feature maps from input images. It leverages the Xception architecture and incorporates the Atrous Spatial Pyramid Pooling (ASPP) module. ASPP plays a critical role in capturing features across various scales and aggregating contextual information from multiple scales. It is composed of parallel dilated convolutions with distinct dilation rates. Smaller dilation rates capture finer details, while larger dilation rates enable a wider context understanding. This diverse range of dilation rates proves instrumental in accurately segmenting different regions within an image. Notably, the ASPP module used in DeepLabV3+ integrates a global average pooling layer to better capture global context information. Therefore, the encoder in DeepLabV3+ encompasses the ASPP module, enabling the extraction of features and comprehensive contextual understanding across different regions of the image. Built upon the Xception architecture, it employs depth-wise separable convolutions to generate high-dimensional feature maps, resulting in enhanced precision for image segmentation.

Decoder: The decoder module in DeepLabV3+ takes the low-resolution feature maps generated by the encoder and restores them to the original high resolution. This restoration process is achieved through a technique called upsampling or deconvolution. The upsampling operation enlarges the low-resolution feature maps and brings them to a size comparable to the original image. Then, these upsampled features are combined with corresponding high-resolution features from the original image (a procedure often called “skip-connections”), which helps the model recover detailed spatial information lost during the downsampling in the encoder. This is critical for pixel-level tasks like segmentation, where accurate localization of objects is needed.

Output: The output of DeepLabV3+ is a pixel-wise classification map, where each pixel is assigned a specific class label indicating the class to which it belongs. This enables precise segmentation of objects in the input image.

### 3.2. Data Augmentation for DeepLabV3+ Model Training

**Figure 7:**
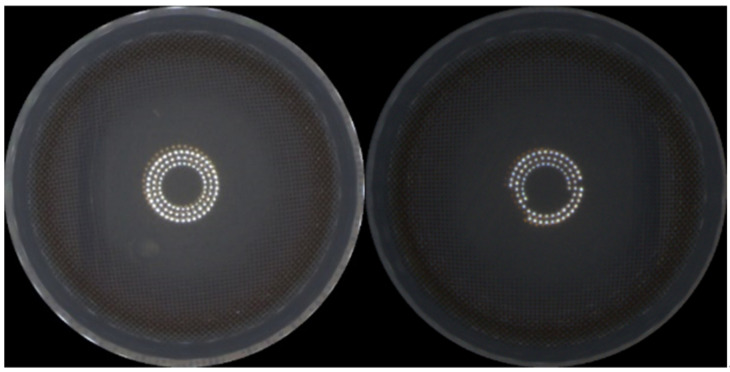
Differences in image brightness caused by external factors such as site lighting, camera lighting, and foreign substances on the camera.

Figure 7 showcases the variations in image data distribution of color contact lenses influenced by various elements such as working conditions, camera brightness, and foreign substances on the camera. Issues related to data bias also arise. To mitigate these, data augmentation techniques were employed, as elaborated below:

In augmenting the training dataset: Images collected from manufacturing environments are subject to variations in brightness due to external factors such as workplace lighting, camera lighting, and foreign substances on the camera lens. These variations distort the image distribution, posing constraints when a deep learning model is trained on limited data. Consequently, predictions for subsequently collected images might not be accurate. To address this, we introduced variability in brightness levels by adding different random values to each RGB (red, green, and blue) channel of the image. This process, termed as “random brightness augmentation”, aids in mimicking different lighting conditions, thereby ensuring the model’s robustness to changes in illumination.

**Figure 8:**
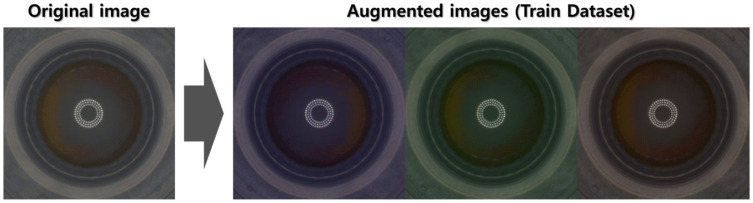
Augmented images using Algorithm 1 (training dataset).

Furthermore, to avert overfitting due to the specific location of features in the image, we employed a “random flip augmentation” technique. Here, the image was randomly flipped left–right or up–down. This augmentation simulates situations where the objects of interest appear at different locations, effectively making the model invariant to the position of these objects. The resulting augmented data were then used as the training dataset. Figure 8 shows an example of augmenting multiple data from a single training data.

Algorithm 1 presents a method to augment image data for our training set. The algorithm operates on an original image (img) over a specified number of iterations, thereby creating a set of augmented images. To begin, it creates an empty list for the augmented images and retrieves the dimensions of the original image. For each iteration, it follows the subsequent steps:

First, it creates a copy of the original image. Then, it generates four random integers. The integer “rand_flip” determines the type of image flip, and the other three integers, namely, “rand_red”, “rand_green”, and “rand_blue”, are used to apply a random brightness adjustment to the respective RGB channels of the image. Depending on the value of “rand_flip”, the copied image is flipped either up–down or left–right.

Subsequently, for each color channel, the algorithm modifies the brightness by adding the respective random value (“rand_red”, “rand_green”, or “rand_blue”) to each pixel, ensuring the pixel values stay within the valid range of 0 to 255 using a clip operation. The augmented image is then appended to the list.

After completing the iterations, the algorithm returns this list of augmented images, which provide a diverse set of training data generated from a single original image.
**Algorithm 1:** AugmentTrainSet(img, iteration, augmented_images)//Augment train images from an original image**Input**(1) img: Original image in RGB channel
(2) iteration: The number of iterations to perform
**Output**(3) augmented_images: List of augmented images
**Begin**1.   augmented_images←emptylist2.   n←heightofimg
3.   m←widthofimg
4.   For i=1 toiteration do
5.   ${img}_{copied}\;\leftarrow \;copy\;of\;img$
6.   ${rand}_{flip}\leftarrow \;random\;integer\;in\;the\;range\;-1\;to\;1$
7.   ${rand}_{red},\;{rand}_{green},\;{rand}_{blue}\;\leftarrow \;random\;integer\;in\;the\;range\;-10\;to\;10$
8.   $If\;{rand}_{flip}\;is\;-1\;then$
9.   ${img}_{copied}\leftarrow img\left[n-i,m-j\right]\;for\;all\;i\;in\;1\;to\;n\;and\;j\;in\;1\;to\;m$
10. $Else\;if\;{rand}_{flip}\;is\;0\;then$
11. ${img}_{copied}\leftarrow img\left[i,m-j\right]\;for\;all\;i\;in\;1\;to\;n\;and\;j\;in\;1\;to\;m$
12. $Else\;if\;{rand}_{flip}\;is\;1\;then$
13. ${img}_{copied}\leftarrow img\left[n-i,j\right]\;for\;all\;i\;in\;1\;to\;n\;and\;j\;in\;1\;to\;m$
14. $For\;each\;pixel\;p\;in\;red\;channel\;of\;{img}_{copied}$
15. $p\leftarrow clip(p+{rand}_{red},\;0,\;255)$
16. End for
17. $For\;each\;pixel\;p\;in\;green\;channel\;of\;{img}_{copied}$
18. $p\leftarrow clip(p+{rand}_{green},\;0,\;255)$
19. End for
20. $For\;each\;pixel\;p\;in\;blue\;channel\;of\;{img}_{copied}$
21. $p\leftarrow clip(p+{rand}_{blue},\;0,\;255)$
22. End for
23. $augmented\_images.append({img}_{copied})$
24. End for
25. Return augmented_images**END**

**Figure 9:**
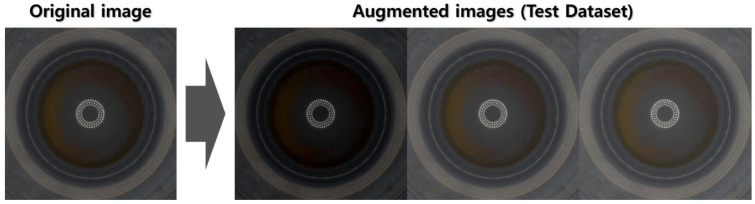
Augmented images using Algorithm 2 (test dataset).

In augmenting the test dataset: While different random values were added to the R, G, and B channels in the training dataset, the same random value was added to the R, G, and B channels of the original image in the test dataset. This is to simulate the potential variability in image brightness caused by external factors. Additionally, image flip was not performed on the test dataset to maintain consistency with real-world scenarios where images are typically not flipped. It also allows us to fairly evaluate the model’s ability to generalize the patterns learned from the training dataset to new data. Algorithm 2 describes the method in detail, with the augmentation results shown in Figure 9.

Algorithm 2 depicts a procedure to augment an image from the test set. The algorithm operates on a single original image (img) and produces one augmented image. Initially, it creates a copy of the original image. Then, it generates a single random integer, “rand_ch”, which will be used for a global brightness adjustment across all RGB channels of the image. This random integer is drawn from the range of −25 to 25. Subsequently, for each pixel in the copied image, the algorithm adds “rand_ch” to the pixel’s value. A clip operation is used to ensure the resulting pixel values remain within the valid range of 0 to 255. The resulting image, which is now the augmented image, is then returned by the algorithm. As a result, we get an augmented test image with a uniformly adjusted brightness level, aiding in evaluating the robustness of the model against varying brightness conditions.
**Algorithm 2:** AugmentTestSet(img, augmented_image)//Augment test images from an original image**Input**(1) img: An original image in RGB channel
**Output**(2) augmented_image: An augmented image**Begin**1. ${img}_{copied}\;\leftarrow \;copy\;of\;img$
2. ${rand}_{ch}\leftarrow \;random\;integer\;in\;the\;range\;-25\;to\;25$
3. $For\;each\;pixel\;p\;in\;{img}_{copied}$
4. $p\leftarrow clip(p+{rand}_{ch},\;0,\;255)$
5. $augmented\_image={img}_{copied}$
6. **End for**
7. Return augmented_image
**End**

### 3.3. Computing the Center Coordinates and Radii of FA and CA Using HCT

**Figure 10:**
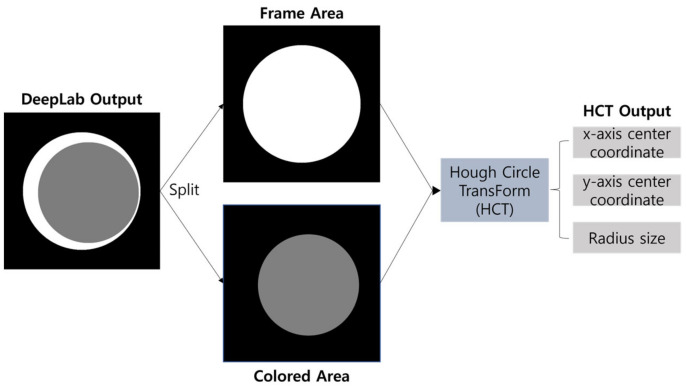
Flowchart of calculating center coordinates and radii using HCT after separating CA and FA from the segmented image.

After splitting the segmented FA and CA areas obtained through DeepLabV3+ into separate images (as shown in Figure 10), the HCT algorithm is employed for calculating the center coordinates and radii. The HCT algorithm is commonly used for circle detection in images. The prior segmentation achieved by DeepLabV3+, which provides distinct FA and CA regions, partitions the image effectively, reducing noise interference and improving the accuracy of circle detection during the subsequent HCT process.

The HCT algorithm proceeds as follows:

Preprocessing: First, the input image is converted to grayscale and then smoothed using Gaussian blur to reduce noise. Subsequently, the canny edge detection algorithm is utilized to identify the edges associated with the circular shapes.

Circle detection: Next, the HCT generates a set of potential circle centers by considering various combinations of edge points and radii. For each edge point and radius, the algorithm calculates all possible circle centers and accumulates them in the Hough space. During this process, the accumulator is incremented at the corresponding pixel positions that intersect with the circle centers.

Circle selection: After the accumulation process, the Hough space is analyzed to determine the most prominent circle centers. The centers with the highest votes are considered as potential circle centers. Finally, the algorithm calculates the corresponding radii for these centers.

In summary, the HCT detects circles by performing preprocessing steps such as grayscale conversion and edge detection, generating potential circle centers through accumulation in the Hough space, and selecting the most prominent circle centers based on votes. By determining the circle centers and radii, this algorithm accurately identifies circles within the image.

In the evaluation metrics, we measure the CD depending on the parameter param1 of the HCT. The HCT algorithm (Algorithm 3) is as follows: **Algorithm 3:** CalculatingHCT(img, dp, minDist, param1, param2, minRadius, maxRadius, $center_{x},\;$
$center_{y},\;radius$)//Detecting a circle to calculate the center of both CA and FA using HCT**Input**(1)   img: an input image
(2)   dp: The inverse ratio of the accumulator resolution to the image resolution
(3)   minDist: Minimum distance between the centers of the detected circles
(4)   param1: 
The higher threshold of the two passed to the Canny edge detector
(5)   param2: 
Accumulator threshold for the circle centers at the detection stage
(6)   minRadius: Minimum circle radius
(7)   maxRadius: Maximum circle radius
**Output**(8)   $center_{x}:\;x\;coordinate\;of\;the\;first\;circle’s\;center$
(9)   $center_{y}:\;y\;coordinate\;of\;the\;first\;circle’s\;center$(10) radius: radius of the first circle
**Begin**1.    gray←ConvertToGrayscale(img)
2.    blured←ApplyGaussianBlur(gray)
3.    edges←ApplyCannyEdgeDetection(blurred, param1)
4.    h, w←get the height and width of ‘edges’5.    accumulator←create a 3D zero array of size (h, w, maxRadius − minRadius)6.    $For\;each\;edge\;point\;\left(x_{edge},\;y_{edge}\right)\;in\;‘edges’\;do$
7.    $For\;r\;in\;range\left(minRadius,\;maxRadius\right)\;do$
8.    $For\;theta\;in\;range\left(0,\;360\right)\;do$
9.    $a\leftarrow x_{edge}-r\;*\cos\left(theta\right)$
10.  $b\leftarrow y_{edge}-r\;*\sin\left(theta\right)$11.  If a and b are within the image boundaries then12.  $accumulator\left[a,\;b,\;r-minRadius\right]\leftarrow accumulator\left[a,\;b,\;r-minRadius\right]\;+\;1$
13.  End for
14.  End for
15.  End for
16.  max_accumulator_value←find the maximum value in ‘accumulator’
17.  circles$\leftarrow find\;the\;\left(a,\;b,\;r\right)\;such\;that\;accumulator\left[a,\;b,\;r\right]>param2\;*\;max\_accumulator\_value$
18.  $center_{x},\;center_{y},\;radius\leftarrow circles\left[0\right]$
19.  $Return\;center_{x},\;center_{y},\;radius$
**END**

### 3.4. Measurement of Center Deviation

**Figure 11:**
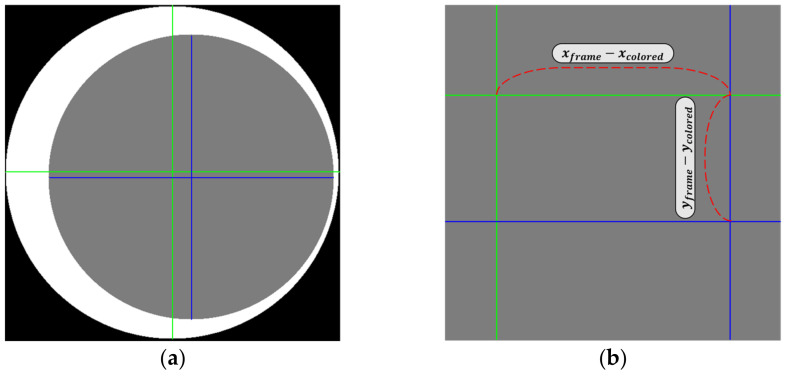
(**a**) Center points of CA and FA in segmented image and (**b**) an enlarged area of the cross-rectangular region. In this figure, the blue line represents the central axis of the CA, and the green line represents the central axis of the FA.

Let $\left(x_{Colored},\;y_{Colored}\right)$ and $\left(x_{Frame},\;y_{Frame}\right)$ represent the center coordinates of CA (gray area in Figure 11a) and FA (white area in Figure 11a), respectively, as calculated using HCT. The intersection point of the vertical and horizontal green lines (blue lines) corresponds to the center of FA (resp. CA), as shown in Figure 11a. Figure 11b shows an enlarged area of the cross-rectangular region.

The CD is determined separately as the center deviation for each of the x-axis and y-axis, with the formula given as follows:

(1) $$Center\;Deviation(CD)=\left(\left|x_{Frame}-x_{Colored}\right|,\left|y_{Frame}-y_{Colored}\right|\right)$$

## 4. Experimental Results

### 4.1. The Number of Datasets

In our study, we used the dataset consisting of a total of 2440 original images. As shown in Table 1, the distribution of images across different lens types was highly skewed, with Lens2 and Lens4 having 1194 and 632 images, respectively, while Lens3 and Lens8 had only 6 and 21 images, respectively. This imbalance in data distribution poses a challenge when dividing the data into training, validation, and testing sets, as it may lead to a lack of learning opportunities for certain lens types and increase uncertainty in real-world predictions.

To improve our model’s predictive reliability for potential variations in image distributions due to internal influences such as lens types and lens position, and external influences such as the working environment, camera brightness, and camera contamination, we implemented an augmentation process for the datasets. This augmentation process in the training set, executed via Algorithm 1, enhanced the original images by adding unique random values to each RGB channel individually and conducted numerous random flips. The “iterations” in Table 1 indicate the number of times augmentation was performed per original image.

The original dataset was chosen as the validation set to verify our model’s generalization capabilities, assessing the augmented training data’s predictive accuracy on actual, unmodified images. This approach was taken to ensure that the model could effectively handle real-world data, which may not always be perfectly balanced or free from noise.

Lastly, we augmented the testing set using Algorithm 2, in which the same random value was added to the RGB channels of the original images. This step was taken to assess our model’s resilience and performance when predicting potential future data influenced by internal and external factors.

As emphasized by Bianco et al. [25], it’s crucial to ensure that the pixel distribution of images remains unchanged due to different lighting conditions during training. In our study, we adopted this principle but used it to enhance diversity. We augmented the training and testing sets from the original images by randomly transforming pixel values and generating noise through random rotations. This process created distinct pixel distributions for the training, validation, and testing sets, allowing us to train and evaluate our model on datasets with various characteristics that may occur in future real manufacturing environments.

**Figure 12:**
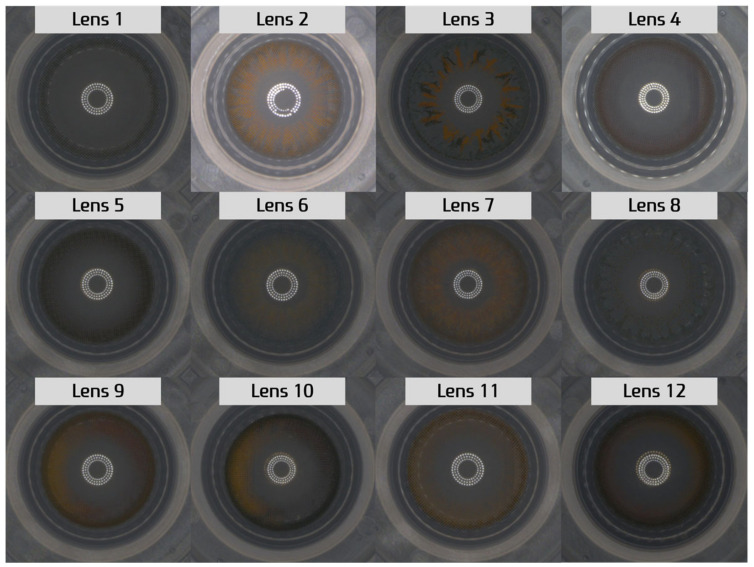
Lens type (original images).

**Figure 13:**
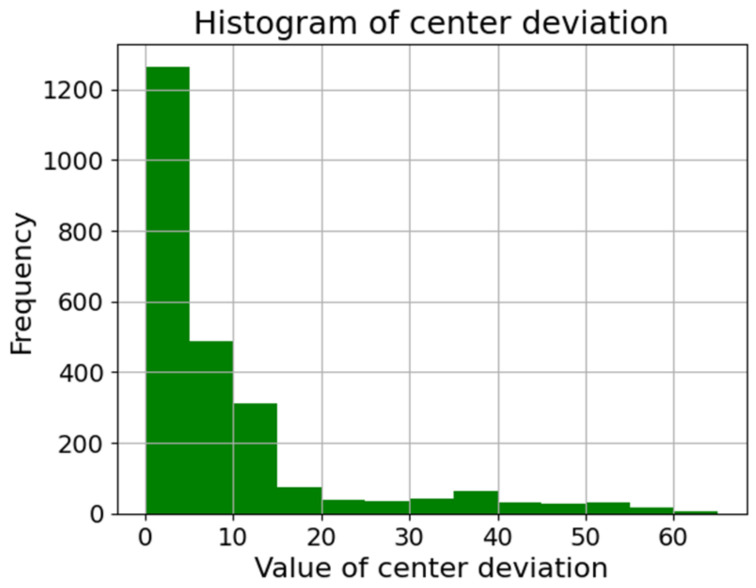
Histogram of the $\left\|CD\right\|$ from the original dataset.

As shown in Figure 12, we used a total of 12 distinct types of lenses in this study. Table 1 outlines the number of images per dataset. Additionally, Figure 13 represents the distribution of the L2 distance (Euclidean distance) for the x and y axes of the CD ($\left\|CD\right\|$) from the original images. Most data points fall within the $\left\|CD\right\|$ interval of 0~5, with the subsequent substantial frequencies found within the $\left\|CD\right\|$ ranges of 5~10 and 10~15, constituting the second and third most prevalent distributions, respectively.

### 4.2. DeepLabV3+ Training Parameters and Prediction Evaluation Results

**Figure 14:**
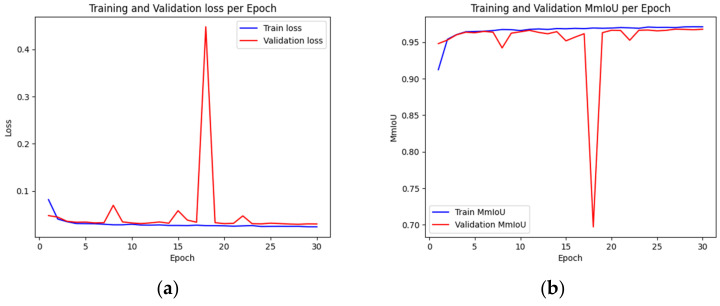
(**a**) Shows the training and validation losses per epoch, and (**b**) shows the training and validation MmIoU per epoch.

In this study, we utilized the DeepLabV3+ model for semantic segmentation of the input images. The training settings outlined in Table 2 were selected based on empirical data, hardware constraints, and the default settings of the DeepLabV3+ model. To avoid GPU memory exhaustion, the batch size was set to eight, and the number of epochs was set to 30. This setting was chosen as the performance plateaued with no significant improvements in both the training and validation losses, and as shown in Figure 14, it was sufficiently trained by this epoch count. The learning rate and the upsampling interpolation method followed the DeepLabV3+ model’s default settings, which have been proven to provide satisfactory results in numerous semantic segmentation tasks. The optimizer was chosen based on its proven efficacy and compatibility with the DeepLabV3+ model.

The mean intersection over union (mIoU) is a prevalent metric in semantic segmentation tasks. It quantifies the overlap between the predicted and ground truth regions for each class, and is computed as the mean of Intersection over Union (IoU) scores per class. The IoU is the ratio of the intersection to the union of the predicted and ground truth regions. In this study, the weights of the model were evaluated based on when the mean of the mIoU (MmIoU) of the validation dataset was the highest during the training process. The formula for the MmIoU is as follows:

(2) $$MmIoU=\frac{1}{N}\sum_{i=1}^{N}{\left(\frac{1}{M}\sum_{j=1}^{M}\frac{TP_{j}}{TP_{j}+FP_{j}+FN_{j}}\right)}_{i}$$

In this formula, N represents the number of images, i represents the i-th image, M represents the number of pixels, and j represents the j-th pixel of an image. TP represents the number of pixels that are correctly predicted as belonging to a class, FP represents the number of pixels that are incorrectly predicted as belonging to a class, and FN represents the number of pixels that are incorrectly predicted as not belonging to a class.

Table 3 presents the MmIoU for the training, validation, and test datasets at the point during the model’s training process when the MmIoU was highest for the validation dataset. In Figure 14, we present two distinct plots: (a) illustrates the trend of loss per epoch, while (b) graphically depicts the evolution of MmIoU across the epochs.

### 4.3. Evaluation Results for CD Prediction

The center coordinates of the CA and FA predicted by our proposed method may not perfectly match the ground truth of centers. Moreover, in cases where the predicted location has no direct relation with the measured location, the accuracy of the prediction may not be precisely reflected by the CD alone. Therefore, we aim to validate the accurate prediction of the center coordinates along with the evaluation based on CD.

In the evaluation formula and metrics, the hat symbol ($\hat{\;}$) represents the predicted value, and its absence indicates the ground truth value. Additionally, the bar symbol ($\bar{\;}$) in evaluation metrics represents the average.

For the center coordinates of the CA and FA, defined as $\left(x_{CA},\;y_{CA}\right)$ and $\left(x_{FA},y_{FA}\right)$, respectively, we evaluate the following error metrics ${Ex}_{CA}$, $Ex_{FA}$, $Ey_{CA}$, and $Ey_{FA}$. The formulas are as follows:

(3) $${Error\;of\;x\;axis\;in\;CA\;(Ex}_{CA})=\left|x_{CA}-{\hat{x}}_{CA}\right|\;{Error\;of\;x\;axis\;in\;FA\;(Ex}_{FA})=\left|x_{FA}-{\hat{x}}_{FA}\right|\;{Error\;of\;y\;axis\;in\;CA\;(Ey}_{CA})=\left|y_{CA}-{\hat{y}}_{CA}\right|\;{Error\;of\;y\;axis\;in\;FA(Ey}_{FA})=\left|y_{FA}-{\hat{y}}_{FA}\right|$$

The mean of the following formulas represents the L_2_ norm (Euclidean distance) of the mean colored area error (MCE) and mean FA error (MFE) used as evaluation metrics for the predicted and actual values in a two-dimensional coordinate system for both the CA and FA. The detailed formulas are as follows:

(4) $$\left\|MCE\right\|=\frac{1}{N}\sum \sqrt{{{Ex}_{CE}}^{2}+{Ey_{CE}}^{2}}\;\left\|MFE\right\|=\frac{1}{N}\sum \sqrt{{Ex_{FE}}^{2}+{{Ey}_{FE}}^{2}}$$

The following formulas represent the mean center deviation error (MCDE), which is the average difference between the predicted CD and the actual CD of the sampled data. The detailed formulas are as follows:

(5) $$x_{MCDE}=\left|\left|x_{CA}-x_{FA}\right|-\left|{\hat{x}}_{CA}-{\hat{x}}_{FA}\right|\right|\;y_{MCDE}=\left|\left|y_{CA}-y_{FA}\right|-\left|{\hat{y}}_{CA}-{\hat{y}}_{FA}\right|\right|\;\left\|MCDE\right\|=\frac{1}{N}\sum \left|\sqrt{{\left(x_{CA}-x_{FA}\right)}^{2}+{\left(y_{CA}-y_{FA}\right)}^{2}}\;\;\;\;\;\;\;\;\;\;\;\;\;\;\;\;\;\;\;\;\;\;\;\;\;\;\;\;\;\;\;\;\;\;\;\;\;\;-\sqrt{{\left({\hat{x}}_{CA}-{\hat{x}}_{FA}\right)}^{2}+{\left({\hat{y}}_{CA}-{\hat{y}}_{FA}\right)}^{2}}\right|$$

In this study, we compared our proposed method, which utilized the weights achieving the highest MmIoU on the validation dataset, solely with the approach proposed by Kim et al. [20]. Kim et al.’s study is the only work that specifically addresses the measurement of lens centroid deviation akin to our research objective and uses the same lens dataset, thereby making it an ideal benchmark for our study. While we appreciate the merits of a broader comparative evaluation, the specificity of our research focus and data constraints have led us to use Kim et al.’s method as the sole point of comparison.

To conduct the comparison of these two methods, we used the test dataset. The results are presented in Table 4. Both “*p*1(param1)” and “*p*2(param2)” in the table are parameters of the HCT, and the evaluation metric values remain unchanged with any variation in “*p*2”. Their definitions can be referenced in Algorithm 3 discussed earlier. The rationale behind this exclusive comparison lies in the unique focus of our research.

Table 4 demonstrates that the proposed method outperformed the method by Kim et al. [20] in terms of lower errors across all evaluation metrics, with the lowest error rate for the Kim et al. method observed at p1=40. Furthermore, the $\left\|MCDE\right\|$ of the proposed method decreased by 71.2% compared with the Kim et al. [20] method at p=40. This can be attributed to the effective noise reduction in the original images using DeepLabV3+ in the proposed method, leading to more accurate predictions of the center coordinates of the CA and FA during the HCT process.

### 4.4. Extension of CD Prediction: Classification of Defects and Normal

The criteria for CD defects in color contact lenses are defined as follows: The CD along the *x*-axis or *y*-axis is 0.4 mm or more, as shown in Formula 6, with the radius ($r_{frame}$) of the FA being 21 mm. The formula for converting the 0.4 mm criteria for CD defects into pixel units is depicted in (7).

(6) $$Center\;deviation\;defects=\max\left(\left|x_{frame}-x_{colored}\right|,\left|y_{frame}-y_{colored}\right|\right)\;\geq Criteria\;for\;center\;defects(pixel)$$

(7) $$Criteria\;for\;center\;defects\left(pixel\right)=\frac{0.4\;mm}{21\;mm}\times r_{frame}\times 2$$

**Figure 15:**
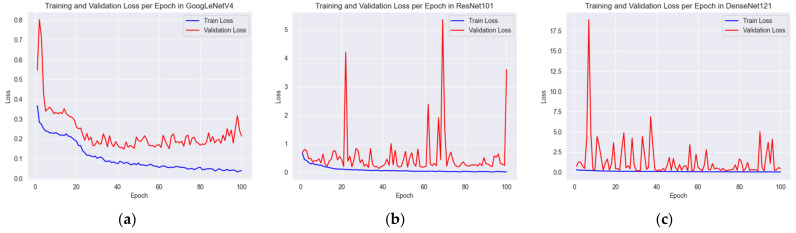
Losses per epoch for each model: (**a**) GoogLeNetV4, (**b**) ResNet101, and (**c**) DenseNet121.

In Section 4.3, we addressed the regression problem of CD and compared our results with the research conducted by Kim et al. [20]. In this part of the study, where we extend our research, we aim to carry out the classification of center deviation defects. For the sake of comparison, we will consider not only the work of Kim et al. [20], but also a different study by the same author, Kim [12], that dealt with defective classification of color contact lenses. Kim [12] used various CNNs to classify the defects of color contact lenses, and we intend to make our comparison using the three models that yielded the best results in that study, namely, GoogLeNetV4 [26], ResNet101 [24], and DenseNet121 [27] in our datasets. The learning configuration of the models is the same as in the previous study [12]. The results of classifying defects based on center deviation criteria in pixel units are given in Table 5. Furthermore, Figure 15 depicts the loss values according to epochs for GoogLeNetV4, ResNet101, and DenseNet121. Upon review, it can be inferred that each of these models has been adequately trained in validation dataset.

As indicated in Table 5, although our proposed method shows the highest accuracy, it does not yield the highest values for precision, recall, and F1-score. However, it is worth noting that the classification in our method is based on measuring the L2 distance of the center deviation (CD). In the actual manufacturing process of color contact lenses, the need to adjust the machines that print the colored area is as significant as defect classification. Therefore, the measurement of CD becomes indispensable. Furthermore, considering the importance of CD measurement for machinery adjustment, it becomes apparent that the value of our method extends beyond mere classification accuracy and offers practical utility in real-world scenarios.

## 5. Concluding Remarks

### 5.1. Summary

In this study, we proposed a method for measuring the CD of color contact lenses using HCT after reducing image noise using DeepLabV3+ trained on augmented original images. This approach created a training dataset by augmenting the original images, allowing our model to learn from a variety of cases and predict images with potential distributions that have not yet been observed. The MCDE of our method showed a 71.2% reduction compared with Kim et al. [20], suggesting that our proposed method could provide an effective solution for measuring the CD of color contact lenses.

### 5.2. Discussion and Future Work

Furthermore, we introduced the concept of center deviation error (CDE) as the difference between the predicted CD and the actual CD. This measure was particularly helpful to gauge the performance of our method under different scenarios. Let the center coordinates of the CA and FA be $\left(x_{CA},\;y_{CA}\right)$ and $\left(x_{FA},y_{FA}\right)$, respectively, with $\left({\hat{x}}_{CA},\;{\hat{y}}_{CA}\right)$ and $\left({\hat{x}}_{FA},{\hat{y}}_{FA}\right)$ being the predicted results of CA and FA, respectively. The CDE is defined as follows:

(8) $$CDE=\left|\sqrt{{\left(x_{CA}-x_{FA}\right)}^{2}+{\left(y_{CA}-y_{FA}\right)}^{2}}-\sqrt{{\left({\hat{x}}_{CA}-{\hat{x}}_{FA}\right)}^{2}+{\left({\hat{y}}_{CA}-{\hat{y}}_{FA}\right)}^{2}}\right|$$

**Figure 16:**
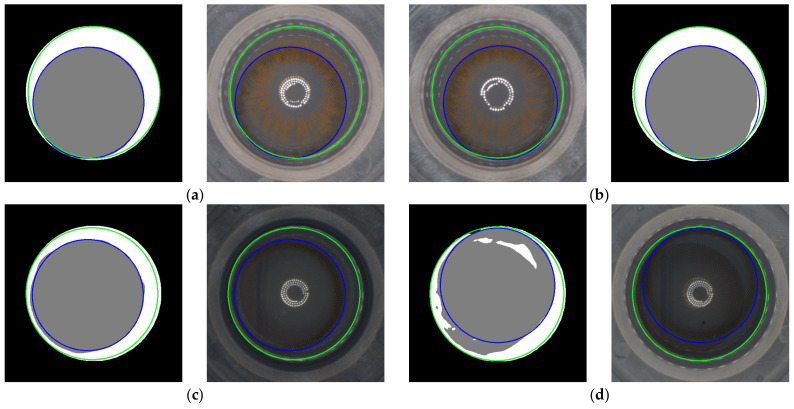
Four cases that had the highest CD error (CDE) predicted using the proposed method on the original dataset. (**a**) CDE: 27.664; (**b**) CDE: 11.416; (**c**) CDE: 12.245; (**d**) CDE: 17.443.

However, there were certain limitations observed in our method. In some cases, the segmentation process via DeepLabV3+ was not successful. As can be seen in Figure 16, the CDE tended to be higher when the CA was divided into two separate areas rather than being composed of a single area. These limitations suggest that our method might encounter difficulties in dealing with more complex or unexpected scenarios.

In our future work, we first aim to minimize the CD by improving our proposed method, employing post-processing techniques, and optimizing the predicted image from DeepLabV3+ to more closely resemble a perfect circle. This would be particularly beneficial in handling images where CA is segmented into multiple areas, thereby enhancing the effectiveness of our CD measurement approach. By considering the results of Jin et al. [28] and Zhao et al. [29], subsequently, we plan to address the learning challenges in new domains by leveraging the powerful techniques of deep learning, namely, transfer learning and domain adaptation. These methods effectively transfer and apply the knowledge learned from existing source domains to new target domains, enhancing performance even when learning in specific domains is challenging. Based on the successful outcomes of these studies, we anticipate that these methods will significantly contribute to solving learning problems in new domains.

## Figures and Tables

**Table 1:** The number of lens datasets.

Lens Type	The Number of Images in Original Dataset	The Number of Images in Training Dataset Augmented by Algorithm 1 (*Original Dataset* × *Iterations*)	The Number of Images in Validation Dataset	The Number of Images in Test Dataset Augmented by Algorithm 2
Lens 1	41	205 (41×5)	41	41
Lens 2	1194	1194 (1194×1)	1194	1194
Lens 3	6	198 (6×33)	6	6
Lens 4	632	632 (632×1)	632	632
Lens 5	193	193 (193×1)	193	193
Lens 6	72	216 (72×3)	72	72
Lens 7	51	204 (51×4)	51	51
Lens 8	21	210 (21×10)	21	21
Lens 9	32	192 (32×6)	32	32
Lens 10	51	204 (51×4)	51	51
Lens 11	50	200 (50×4)	50	50
Lens 12	97	194 (97×2)	97	97
Total	2440	3842	2440	2440

**Table 2:** DeepLabV3+ training settings.

Epoch	30
Optimizer	Adam
Batch size	8
Learning rate	0.007
Image size	Width: 512, height: 512, channel: 3 (RGB)
Preprocessing	Min–Max scaling
Kernel initialization	HeNormal
Upsampling interpolation	Bilinear interpolation

**Table 3:** MmIoU results obtained from the deep learning model.

Dataset	MmIoU
Training dataset	0.9723
Validation dataset	0.9675
Test dataset	0.9660

**Table 4:** Evaluation metrics (unit: pixels).

Method	p1	Image Size	*x* and *y* Axis Error				CA and FA Error		CD Error		
			${\bar{Ex}}_{CA}$	${\bar{Ey}}_{CA}$	${\bar{Ex}}_{FA}$	${\bar{Ey}}_{FA}$	‖MCE‖	‖MFE‖	${\bar{x}}_{MCDE}$	${\bar{y}}_{MCDE}$	$\left\|MCDE\right\|$
Proposal method	1	512	1.329	2.639	1.373	1.358	3.314	2.101	1.592	2.766	2.902
Kim et al. [20]	80		7.083	8.491	4.06	4.003	12.018	6.097	8.295	9.557	12.993
	70		5.6	7.212	4.808	4.727	9.974	7.182	7.922	9.102	12.378
	60		4.242	5.929	5.139	5.009	8.02	7.619	7.277	8.227	11.168
	50		2.735	4.536	5.775	5.778	5.885	8.639	6.563	7.948	10.414
	40		2.161	3.89	6.293	6.101	4.976	9.23	6.552	7.676	10.075
	30		2.364	4.106	10.955	11.205	5.297	16.273	11.131	12.475	16.93
	20		2.847	4.536	23.842	24.137	6.013	34.596	23.304	24.576	34.178
	10		4.998	5.918	34.81	35.495	8.792	50.856	33.325	34.662	48.911

**Table 5:** Evaluation metrics for CD defects classification from test dataset.

Method	Accuracy	Precision	Recall	F1 Score
Proposal method	0.9298	0.7853	0.8712	0.826
Kim et al. [20] (*p*1 = 40)	0.6672	0.3568	0.9277	0.5147
GoogLeNetV4	0.8445	0.8812	0.9335	0.9066
ResNet101	0.8683	0.9192	0.9178	0.9185
DensNet121	0.8732	0.9416	0.899	0.9198

## Data Availability

Access to the data is restricted due to proprietary constraints enforced by the data-holding enterprise. Therefore, it is not available for use upon request.
